# Associations of personality traits with internet addiction in Chinese medical students: the mediating role of attention-deficit/hyperactivity disorder symptoms

**DOI:** 10.1186/s12888-019-2173-9

**Published:** 2019-06-17

**Authors:** Meng Shi, Tian Jiao Du

**Affiliations:** 1 Department of English, School of Fundamental Sciences, China Medical University, 77 Puhe Road, Shenyang North Development Zone, Shenyang, 110122 People’s Republic of China; 2 Department of Psychology, School of Humanities and Social Sciences, China Medical University, 77 Puhe Road, Shenyang North Development Zone, Shenyang, 110122 People’s Republic of China

**Keywords:** Personality traits, Internet addiction, ADHD symptoms, Medical students

## Abstract

**Background:**

Internet addiction (IA) has emerged as a public health concern, particularly among adolescents and young adults. However, few studies have been conducted in medical students. This multi-center study aimed to investigate the prevalence of IA in Chinese medical students, to examine the associations of big five personality traits with IA in the population, and to explore the possible mediating role of attention-deficit/hyperactivity disorder (ADHD) symptoms in the relationship.

**Methods:**

Self-reported questionnaires, including Internet Addiction Test (IAT), Big Five Inventory (BFI), Adult ADHD Self-Report Scale-V1.1 (ASRS-V1.1) Screener, and socio-demographic section were distributed to clinical students at 3 medical schools in China. A total of 1264 students became the final subjects.

**Results:**

The overall prevalence of IA among Chinese medical students was 44.7% (IAT > 30), and 9.2% of the students demonstrated moderate or severe IA (IAT ≥ 50). After adjustment for covariates, while conscientiousness and agreeableness were negatively associated with IA, neuroticism was positively associated with it. ADHD symptoms mediated the associations of conscientiousness, agreeableness and neuroticism with IA.

**Conclusion:**

The prevalence of IA among Chinese medical students is high. Both personality traits and ADHD symptoms should be considered when tailored intervention strategies are designed to prevent and reduce IA in medical students.

## Background

The past two decades has witnessed a tremendous growth of global Internet users, with the figure increasing substantially from 0.4 billion in 2000 to 4.2 billion in 2018, and half of its current users are located in Asia [1]. It is widely acknowledged that Internet has brought huge benefits to individuals, organizations and society, like higher accessibility of information, and more communication and entertainment options. However, excessive use of Internet could lead to Internet addiction (IA) [2, 3], characterized by one’s inability to inhibit Internet use despite negative effects on many domains of life, such as academic performance, social relations, physical and mental health, and quality of life [2, 4–7].

IA has emerged as an important issue in the fields of public health and psychiatry. Given the psychological and developmental characteristics, university students are particularly susceptible to IA [8]. Recent studies have revealed that the prevalence of IA among college students varies significantly, ranging from 3.2% in British students, to 16.3% in Italian students, and 21.2% in Chinese students [9–11]. This wide difference in prevalence can partly be attributed to different assessment instruments. With regard to medical students, a latest meta-analysis has demonstrated that the pooled prevalence of IA among medical students in 6 countries is up to 30.1% [12], which is five times that of the general population [5]. Due to the stress inherent in medical education, many students are vulnerable to psychological and psychiatric disorders, such as depression and anxiety [13], which are positively associated with IA [14]. Though preliminary evidence has shown that the prevalence of IA in medical students may not be significantly different from that of other student groups [15–17], it is also revealed that the prevalence may not drop when the students become junior physicians [17, 18]. As IA is associated with cognitive impairment [5], quality of care and safety of patients could be negatively affected if no effective intervention strategies are undertaken to deal with the issue of IA among physicians-in-training. Therefore, IA in medical students and its key related factors warrant further investigation.

Personality traits can predict one’s behaviors, and it has been consistently found that they are associated with IA in different populations and cultures [10, 19, 20]. The Five-Factor Model (FFM) is the most established personality model, which recognizes that personality traits are hierarchically organized into five broad dimensions, consisting of extraversion, neuroticism, conscientiousness, agreeableness, and openness [21]. FFM is determined by biological factors, and transcends languages and cultures [22]. A meta-analytic review of big five personality traits and IA has demonstrated that all the five dimensions are significantly related to IA. While neuroticism is positively associated with IA, all the other four dimensions are negatively related to it. In terms of effect size, conscientiousness is found to be the strongest predictor, whereas openness is the weakest one [19]. However, it is worth noting that independent studies show large heterogeneity with regard to the relations between big five personality traits and IA. For instance, four dimensions were found to be predictors of IA in Iranian undergraduates with the exception of neuroticism [23]. Agreeableness and extraversion were shown to be negatively associated with IA, while openness was positively associated with it in Italian university students [10]. In Norwegian undergraduates, conscientiousness, neuroticism, and agreeableness were significant predictors [24], but only the first two dimensions were related to IA among Colombian college students [25]. It should be mentioned that after controlling for demographic variables, whereas conscientiousness and agreeableness were negatively related to IA among Chinese adolescents, all the three remaining dimensions were positively related to it [20]. As of yet, the relations between big five personality traits and IA have not been examined in Chinese university students.

Attention-deficit/hyperactivity disorder (ADHD), traditionally considered a childhood disorder, can persist into adulthood for approximately two-thirds of affected children and adolescents [26]. Two core symptoms of ADHD, being bored easily and delay aversion [27, 28], may predispose individuals to indulge in various online activities. For example, a 2-year prospective study of 2293 adolescents in Taiwan showed that ADHD was the leading risk factor for the occurrence of IA out of several psychiatric symptoms [29]. Another study carried out among Turkish university students suggested that severity of ADHD symptoms predicted severity of IA even after controlling for personality traits, depression and anxiety symptoms [30]. The meta-analysis of the associations between IA and psychiatric disorders also revealed that IA had a stronger correlation with ADHD relative to depression and anxiety [14]. Though the association between IA and ADHD is robust, the majority of the studies are conducted in Taiwan and South Korea [31], and this association has neither been examined in college students in mainland of China nor in medical students worldwide, despite the fact that a recent large survey has demonstrated that ADHD is the most common self-disclosed disability for medical students to receive accommodations out of all types of disabilities [32].

In terms of the relations between big five personality traits and ADHD symptoms, the results are mixed. Using different assessment tools for Big Five and ADHD symptoms, Nigg et al. demonstrated that ADHD symptoms were significantly associated with low conscientiousness and agreeableness, as well as high neuroticism [33], which is congruent with the results of the meta-analytic review of the relation between the two constructs [34]. Meanwhile, Big Five were found to account for 41.4% of the variance in ADHD symptoms in a large sample of Canadian university students, with all the five dimensions being significant predictors [35]. While lower extraversion and openness were reported in ADHD patients [36], higher extraversion and lower openness were revealed in university students with ADHD symptoms [35].

Though the associations of ADHD symptoms with personality traits and IA have been examined in previous studies, the possible mediating role of the symptoms in the relation between the two variables has yet been explored. According to the vulnerability model regarding the relations between personality and psychopathology, certain personality traits can predispose individuals to certain kinds of psychopathology, including ADHD symptoms [34]. In addition, the core symptoms of ADHD may predispose individuals to IA. Thus, it was hypothesized that ADHD symptoms might function as a mediator in the relations between big five personality traits and IA. The objectives of the present study were to investigate the prevalence of IA in Chinese medical students, to examine the associations of big five personality traits with IA in this population, and to explore the possible mediating effects of ADHD symptoms on the associations.

## Methods

### Study population and design

From late September to mid-November in 2017, this multi-center cross-sectional study was carried out at three medical schools in different regions of China, including China Medical University, Guizhou Medical University, and Xiangya School of Medicine. Based on academic year, whole classes of clinical students were randomly chosen from each institution, and the students were invited to participate in the survey on a voluntary basis. A total number of 1420 questionnaires were distributed and 1312 were returned. After excluding 48 invalid questionnaires, 1264 students (effective response rate: 89.01%) became the final subjects. The study was approved by the Institutional Review Board of China Medical University, and written informed consents were obtained from the participants according to the Declaration of Helsinki.

### Measurement of internet addiction

The 20-item Internet Addiction Test (IAT) was used to evaluate IA in the students [37]. The IAT is the most widely used measure for IA worldwide and in China [15, 38, 39], and is rated on a 6-point Likert scale, with a total score ranging from 0 to 100. According to the manual, total scores that range from 0 to 30 reflect a normal level of Internet usage; scores of 31 to 49 indicate the presence of a mild level of IA; scores of 50 to 79 reflect the presence of a moderate level, and scores from 80 to 100 indicate a severe dependence upon the Internet [40]. Developed as a unidimensional instrument, the IAT has demonstrated adequate psychometric properties, but its optimal overall structure has yet to emerge [39, 41, 42]. In the present study, the IAT was considered a one factor model, and the Cronbach’s alpha for the scale was 0.916.

### Measurement of personality traits

Personality traits were measured with the 44-item Big Five Inventory (BFI) [43], which covers the five dimensions of personality traits consisting of extraversion, agreeableness, conscientiousness, neuroticism and openness. Each item is scored on a 5-point Likert scale from 1 (disagree strongly) to 5 (agree strongly). The Chinese version of BFI has shown adequate psychometric properties [44, 45]. In this study, the Cronbach’s alpha coefficients for extraversion, agreeableness, conscientiousness, neuroticism and openness were 0.733, 0.688, 0.741, 0.730 and 0.763, respectively.

### Measurement of ADHD symptoms

Adult ADHD Self-Report Scale-V1.1 (ASRS-V1.1) was developed based on the DSM-IV Criterion A symptoms of ADHD [46]. Its 6-item ASRS-V1.1 Screener was found to outperform the 18-item ASRS-V1.1 in terms of sensitivity, specificity and predictive accuracy. Each item is rated on a 5-point Likert scale, with the total score indicating severity of ADHD symptoms and risk for diagnosed ADHD [46, 47]. In the current study, the Cronbach’s alpha for ASRS-V1.1 Screener was 0.680.

### Demographic characteristics

Demographic information regarding age, gender, academic year, and hometowns were obtained in the study. Hometowns were dichotomized into urban area and non-urban area.

### Statistical analysis

All analyses were performed using SPSS 13.0, and the significance level of statistical tests was set at *p* <  0.05. Descriptive statistics of demographic and psychological variables were indicated with mean, standard deviation (SD), number (N) and percentage (%) as appropriate. T-tests and one-way ANOVA were used to compare differences of IA in categorical groups. Pearson’s correlation was used to examine correlations between the continuous variables. Hierarchical regression analysis was performed to explore the effects of groups of independent variables on IA. In step 1, the demographic variables were entered; in step 2, big five personality traits were entered; in step 3, ADHD symptoms were added. Standardized estimate (β), F, R^2^ and R^2^-changes (△R^2^) for each step were provided. Asymptotic and resampling strategies, based on 5000 bootstrap samples, were used to examine the mediating role of ADHD symptoms on the associations of personality traits with IA [48]. The bias-corrected and accelerated 95% confidence interval for each a * b product was calculated to evaluate the mediation effect. A path analysis was also performed using Amos 23.0 to further validate the mediation model. All continuous variables were standardized to avoid multicollinearity before the regression analyses were performed [49].

## Results

### Characteristics of subjects

The demographic characteristics of the medical students and the distribution of IA in categorical variables are shown in Table 1. Among the 1264 students, 520 (41.1%) were males, and 744 (58.9%) were females. Their age ranged from 17 to 26 (M = 19.74, SD = 1.48). The overall prevalence of IA among the medical students was 44.7% (IAT > 30), with 35.5, 8.6, and 0.6% of the students presenting mild (31 ≤ IAT ≤ 49), moderate (50 ≤ IAT ≤ 79), and severe IA (IAT ≥ 80), respectively. There were significant differences of IA in terms of age group (*p* = 0.004), academic year (*p* <  0.001) and hometowns (*p* = 0.046).

**Table 1:** Characteristics of Study Population (*N* = 1264)

Variables	No	%	IAT (Mean ± SD)	P
Gender				
Male	520	41.1%	30.55 ± 15.57	0.232
Female	744	58.9%	29.54 ± 13.66	
Age group				
17–19	610	48.9%	28.75 ± 13.69	0.004
20–26	654	51.7%	31.07 ± 15.10	
Academic year				
1st year	395	31.3%	27.46 ± 13.35	< 0.001
2nd year	329	26.0%	30.42 ± 14.50	
3rd year	263	20.8%	30.76 ± 15.07	
4th year	277	21.9%	32.18 ± 14.99	
Hometown				
Urban area	643	50.9%	30.75 ± 15.39	0.046
Non-urban area	621	49.1%	29.13 ± 13.44	

*IAT* Internet Addiction Test

### Correlations between the variables

The means, standard deviations and the correlations of all the continuous variables are revealed in Table 2. As demonstrated, age was not significantly related to any variable except openness. The traits of extraversion, agreeableness, conscientiousness and openness were all negatively associated with IA and ADHD symptoms, whereas neuroticism was positively associated with both of them. ADHD symptoms were positively related to IA.

**Table 2:** Means, standard deviation (SD) and correlations of continuous variables

Variables	Mean	SD	1	2	3	4	5	6	7
1. Age	19.74	1.48	1						
2. Internet addiction	29.95	14.49	0.045	1					
3. Extraversion	24.51	4.97	−0.040	−0.159^**^	1				
4. Agreeableness	33.84	4.73	−0.043	− 0.315^**^	0.165^**^	1			
5. Conscientiousness	29.49	5.12	0.002	−0.419^**^	0.264^**^	0.342^**^	1		
6. Neuroticism	23.09	5.05	0.045	0.345^**^	−0.367^**^	−0.438^**^	− 0.414^**^	1	
7. Openness	33.79	5.78	−0.058^*^	−0.159^**^	0.415^**^	0.185^**^	0.349^**^	−0.284^**^	1
8. ADHD symptoms	9.05	3.39	0.041	0.462^**^	−0.101^**^	−0.289^**^	− 0.377^**^	0.347^**^	− 0.174^**^

** *p* < 0.01 (two-tailed), * *p* < 0.05 (two-tailed)

### Associations of personality traits and ADHD symptoms with IA

The results of the hierarchical regression of IA are presented in Table 3. While the demographic factors explained only 2.1% of the variance in IA, big five personality traits accounted for 22.2% of its variance. After adjustment for covariates, three dimensions were significantly related to IA. Specifically, both conscientiousness (*β* = − 0.318, *p* < 0.01) and agreeableness (*β* = − 0.123, *p* < 0.01) were negatively associated with IA, whereas neuroticism (*β* = 0.164, *p* < 0.01) was positively associated with it. The effect of ADHD symptoms on IA was significantly positive (*β* = 0.319, *p* < 0.01), explaining an additional 8.0% of the variance.

**Table 3:** Hierarchical linear regression analyses results

Variables	Step 1 (*β*)	Step2 (*β*)	Step3(*β*)
Gender	−0.036	−0.073**	− 0.061*
Age group	−0.007	0.010	0.001
Hometown	−0.062*	−0.045	− 0.079**
Grade group 1	0. 094**	0.054	0.057
Grade group 2	0. 101*	0.038	0.060
Grade group 3	0. 144**	0.103*	0.096*
Extraversion		−0.001	−0.027
Agreeableness		−0.123**	−0.082**
Conscientiousness		−0.318**	−0.232**
Neuroticism		0.164**	0.096**
Openness		0.012	0.015
ADHD symptoms			0.319**
F	4.400**	36.536**	49.660**
R^2^	0.021	0.243	0.323
△R^2^	0.021	0.222	0.080

Grade group 1 = 2nd year/1st year, Grade group 2 = 3rd year/1st year, Grade group 3 = 4th year/1st year

** *p* < 0.01 (two-tailed), * *p* < 0.05 (two-tailed)

### Mediating role of ADHD symptoms on the associations of personality traits with IA

Path coefficients, effect sizes of the mediator (a * b products), and 95% CI for the products are presented in Table 4. Since extraversion and openness were not significantly related to IA both before and after ADHD symptoms were entered (c and c’ paths), they failed to satisfy the condition of mediation. The other three dimensions were significantly associated with ADHD symptoms (a path) and IA (c path), and ADHD symptoms were significantly associated with IA (b path). Thus, ADHD symptoms mediated the associations of conscientiousness (a * b = − 0.085, 95% CI: − 0.110, − 0.066), agreeableness (a * b = − 0.041, 95% CI: − 0.063, − 0.022) and neuroticism (a * b = 0.068, 95% CI: 0.047, 0.093) with IA.

**Table 4:** Mediating role of ADHD on the associations of personality traits with Internet addiction

Predictors	Path coefficients				a * b (95% CI)
	*c*	*a*	*b*	*c’*	
Extraversion	−0.001	0.082**	0.318**	− 0.027	0.026 (0.007, 0.048)
Agreeableness	−0.123**	−0.129**	0.318**	−0.082**	− 0.041 (− 0.063, − 0.022)
Conscientiousness	−0.317**	− 0.269**	0.318**	− 0.231**	−0.085 (− 0.110, − 0.066)
Neuroticism	0.163**	0.214**	0.318**	0.095**	0.068 (0.047, 0.093)
Openness	0.012	−0.009	0.318**	0.015	−0.003 (− 0.022, 0.017)

*c*: associations of personality traits with Internet addiction; *a*: associations of personality traits with ADHD symptoms; *b*: associations of ADHD symptoms with Internet addiction after controlling for the predictor variables; c*’*: associations of personality traits with Internet addiction after adding ADHD symptoms as mediator; a * b: the product of a and b; 95% CI: the bias-corrected and accelerated 95% confidence interval

Gender, age group, hometown, and grade year were covariates. ** *p* < 0.01(two-tailed)

**Fig. 1:**
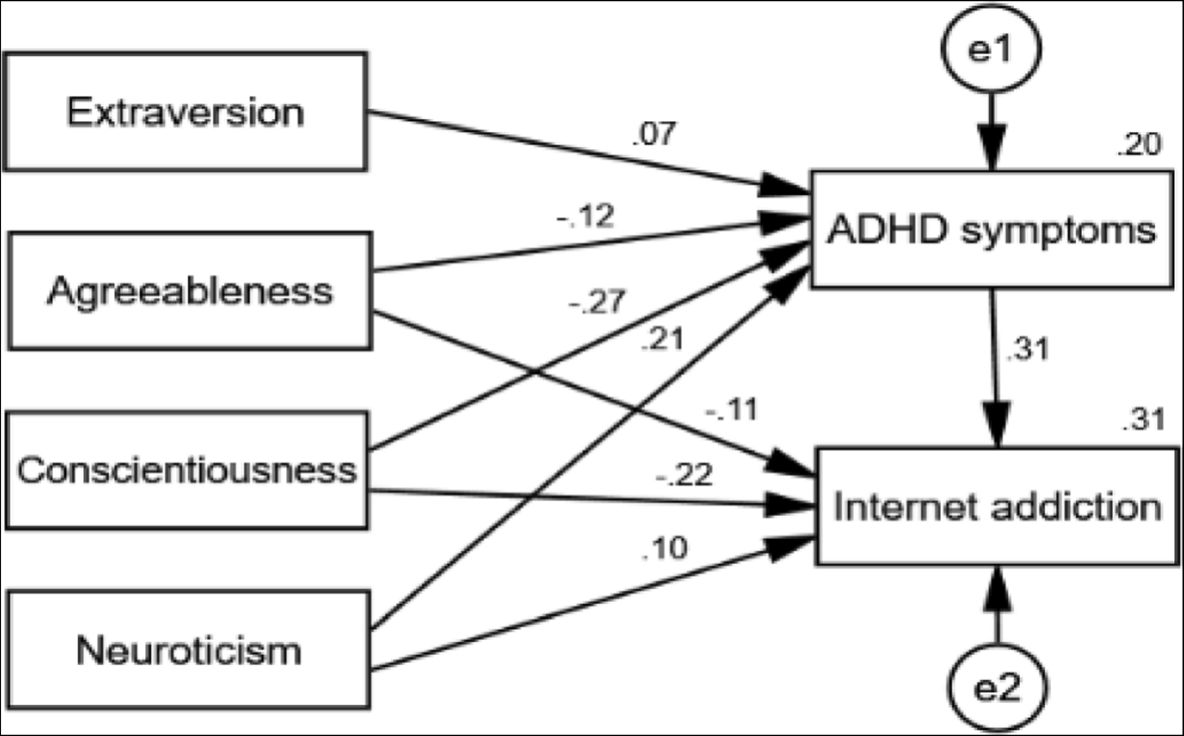
Path analysis depicting direct and indirect effects of personality traits on Internet addiction. Standardized coefficients are presented, and covariates were included in the model but are not presented for simplicity

To further validate the meditational model, a path analysis was performed. Examination of the goodness of fit indicated that the model was fairly adequate (χ^2^/df = 1.227, CFI = 1.000, GFI = 0.999, AGFI = 0.992, RMSEA = 0.013). The results of the path analysis were shown in Fig. 1, which were consistent with those of the regression analysis as well as the asymptotic and resampling strategies.

## Discussion

To the best of our knowledge, this large multi-center study is the first one to examine the associations between big five personality traits and IA in medical students, and to explore the mediating effect of ADHD symptoms on the associations. The study showed that the overall prevalence of IA among Chinese medical students was 44.7%, which was higher than 32.2% in medical students worldwide assessed by the IAT. However, caution should be taken when the prevalence is interpreted and compared since out of dozens of instruments for assessing IA, none has emerged as the diagnostic gold standard [38, 39], and different cutoffs of IAT were utilized in research [12]. Using the cutoff of IAT ≥ 50, the prevalence of 9.2% in the current study was higher than 3.6% in American college students [50], similar to 9.7% in Colombian college students [25], lower than 10.8% in Iranian medical students [51], 11.7% in Chilean medical students [52], and 16.3% in Italian university students [10]. Given the affordability and easy Internet accessibility of smartphones, almost all the university students possess smartphones in China today, and they are often found to use smartphones during lectures, engaging in activities not related to academic study, such as using social media and playing online games. This phenomenon is in line with the finding of a recent study which showed that 95% Brazilian medical students reported using their smartphones inside the classroom for non-medical related activities, and nearly a third of the students used them “always” or “almost always” [53]. The overall prevalence of IA among Chinese medical students was similar to the rate of anxiety symptoms (47.3%) in this population [54], suggesting that IA, as a probable psychological or psychiatric disorder, might be largely neglected in the past, and should be given adequate attention from all parties involved.

After adjustment for demographic factors, three personality traits significantly predicted IA in the students. Based on the absolute value of standardized β, conscientiousness, neuroticism, and agreeableness contributed to the variance in IA. Among the three dimensions, conscientiousness was the strongest predictor, which was correspondent with the result of the meta-analysis of the relations between personality traits and IA [19]. Conscientiousness reflects individual differences in following socially prescribed impulse control that facilitates task- and goal-directed behaviors, such as thinking before acting, planning and organizing tasks, and delaying gratification [43]. High levels of conscientiousness are correlated with reduced exposure to stress, increased appraisal of coping abilities, cognitive restructuring, and higher levels of control in stressful contexts [55, 56]. As conscientiousness-related traits are negatively related to a variety of health-risk behaviors [57], conscientious individuals can choose, create, and evoke healthier environments [58]. They are self-disciplined, diligent and goal striving, so that they can have better control of Internet use, and refrain from becoming addicted to it. In contrast, individuals with low conscientiousness are prone to impulsivity and disorganization, and tend to procrastinate [59, 60]. Internet provides these people opportunities to procrastinate on their tasks and engage in their preferred online activities [61].

The positive association between neuroticism and IA is consistent with the results of many prior studies [19, 24, 25]. Individuals with high levels of neuroticism are characterized by feeling anxious, nervous, sad and tense [43], and they tend to see people and events in a more negative light and are oversensitive to environments. Neuroticism consistently emerges as an important predictor of negative social relations [62]. Compared with less neurotic ones, individuals with high levels of neuroticism are more likely to perceive and receive less social support, and experience more negative interactions within their social networks [63]. Besides, neuroticism also predicts maladaptive coping strategies, such as avoidance, withdrawal, and other emotion-oriented strategies [56]. Thus neurotic individuals may turn to Internet to cope with stress and loneliness facing them in reality.

Agreeableness refers to individual differences in people’s interest in the needs and well-being of others, and is characterized by social adaptability and emotional support [43]. The negative association of agreeableness with IA found in the study is in line with the results of previous research [10, 23, 24]. While agreeable ones are motivated to avoid emotions that may result in interpersonal conflicts [64], less agreeable individuals are more likely to endorse adversarial attitudes towards people around them. Due to the lack of social connections and appropriate social skills, disagreeable people tend to have more disposable time. They may avoid social interactions in reality and turn to Internet to fulfill certain social roles in virtual reality, and thus are more likely to become addicted to Internet than their counterparts.

Personality traits were not only directly associated with IA, but also indirectly associated with it through ADHD symptoms. ADHD symptoms mediated the associations of conscientiousness, agreeableness and neuroticism with IA in the students. Higher scores on conscientiousness and agreeableness were related to lower levels of ADHD symptoms, which in turn were related to lower levels of IA. In contrast, higher scores on neuroticism were associated with higher levels of ADHD symptoms, which were associated with higher levels of IA. While the robust associations of ADHD with personality traits of conscientiousness, agreeableness and neuroticism, as well as IA have been confirmed by prior meta-analyses [14, 34], this study examined the mediating effects of ADHD symptoms on the associations of the three personality traits with IA. Conscientiousness is the personality trait that is most strongly related to ADHD symptoms, and low conscientiousness can predict problems in attention and organization [33]. The core features of neuroticism, feeling anxious and nervous, can interfere with one’s cognition and contribute to ADHD symptoms [34]. Low agreeableness is strongly related to aggressive, intrusive, and delinquent behaviors of ADHD symptoms [33]. Meanwhile, Internet provides multiple opportunities for individuals with ADHD symptoms to meet their psychological needs, and thus they may gradually become addicted.

The implication of the mediation effects is that intervention strategies for IA may consider treatment of ADHD symptoms. Psychosocial treatment is an effective treatment option for adults with ADHD, especially when medication alone fails to work or its side effects are a concern. Cognitive-behavioral therapy, often incorporating modules regarding distractibility, organizational skills and cognitive restructuring, can be tailored to successfully treat ADHD [65–67]. Given that ADHD is the top contributing factor for medical students to receive accommodations [32] and there are high comorbidities of ADHD with other psychiatric disorders [66], well-designed studies are needed to evaluate the intervention effects of tailored cognitive-behavioral therapy to deal with ADHD symptoms in this population.

Several limitations of the study should be acknowledged. First, due to the cross-sectional nature, causality of the involved constructs cannot be determined based on the available data and analysis, and the findings should be confirmed by prospective cohort studies in the future. Second, all data were obtained through self-reported questionnaires, which might introduce response bias. Third, despite the multi-center design, before generalizing the results, more studies should be carried out in other nations where different cultures and medical education systems exist.

## Conclusion

This study revealed that the prevalence of IA among Chinese medical students is high, which warrants more attention from all parties involved. Three dimensions of big five personality traits significantly predict IA in the students. While neuroticism is a risk factor for IA, conscientiousness and agreeableness function as protective factors. ADHD symptoms mediate the associations of the three personality traits with IA. Thus, both personality traits and ADHD symptoms should be taken into account when tailored intervention strategies are designed to prevent and reduce IA in medical students.

## Data Availability

The datasets used and/or analyzed during the current study are available from the corresponding author on reasonable request.

## Abbreviations

ADHD: Attention-deficit/hyperactivity disorder
ASRS-V1.1: Adult ADHD Self-Report Scale-V1.1
BFI: Big Five Inventory
FFM: Five-Factor Model
IA: Internet addiction
IAT: Internet Addiction Test
